# Moderate-to-Heavy Alcohol Consumption May Cause a Significant Decrease in Serum High-Density Lipoprotein Cholesterol in Middle-Aged Women: A Cohort Study of the National Database Study in the Kanto 7 Prefectures-4

**DOI:** 10.7759/cureus.55467

**Published:** 2024-03-04

**Authors:** Airi Sekine, Kei Nakajima

**Affiliations:** 1 Department of Food and Nutrition, Japan Women’s University, Tokyo, JPN; 2 Department of Endocrinology and Diabetes, Saitama Medical Center, Saitama Medical University, Kawagoe, JPN

**Keywords:** alcohol consumption, sex-related differences, artificial intelligence, epidemiology, high-density lipoprotein cholesterol

## Abstract

Aims

Little is known about the association between habitual alcohol consumption and serum high-density lipoprotein cholesterol (HDL-C) in women. We aimed to investigate this association in middle-aged Japanese women in a community-based cohort study using conventional statistical analyses and explainable artificial intelligence (AI) analysis.

Methods

We retrospectively investigated the association between alcohol consumption and HDL-C after 10 years in 90,053 women aged 40-64 years whose drinking habits were generally consistent for 10 years.

Results

After 10 years, 11.3% and 17.9% of subjects had serum HDL-C decreased by ≥10 mg/dL and ≥10%, respectively. In unadjusted analysis, moderate-to-heavy alcohol consumption may both increase and decrease serum HDL-C levels after 10 years. After adjustment for potential confounding factors, moderate (23-45 g/day) and heavy (≥46 g/day) alcohol consumption were each significantly associated with decreases in HDL-C (OR (95% CI): 1.18 and 1.36 (1.11-1.26 and 1.21-1.53) for ≥10 mg/dL, 1.11 and 1.29 (1.05-1.17 and 1.17-1.43) for ≥10%), but not associated with an increase in HDL-C (0.96 and 0.98 (0.91-1.01 and 0.89-1.08) for ≥10 mg/dL, 0.97 and 0.96 (0.93-1.01 and 0.88-1.05) for ≥10%). Further analysis after adjustment for baseline serum HDL-C showed the same results. AI analysis showed that alcohol consumption was the 8th positive contributor to the decrease in HDL-C, following baseline high HDL-C (≥77 mg/dL), high low-density lipoprotein cholesterol (≥133 mg/dL), high body mass index (≥23.1 kg/m^2^), pharmacotherapy for dyslipidemia, high triglycerides (≥70 mg/dL), age 44-64 years, and smoking. Heavy alcohol consumption was a more positive contributor to decreased HDL-C than were other alcohol consumption levels.

Conclusions

Habitual moderate-to-heavy alcohol consumption may cause a significant decrease in serum HDL-C in middle-aged women, which may be modified by concomitant factors.

## Introduction

Although heavy alcohol drinking increases the risk of serious health problems including cardiovascular disease (CVD) [1], cancer [2], cirrhosis of the liver [3], chronic pancreatitis [3], and cardiometabolic diseases such as diabetes, hypertension, and dyslipidemia [4], light-to-moderate alcohol drinking has been associated with decreased risk of CVD such as stroke [5,6] and myocardial infarction [7,8]. Many studies have shown that moderate alcohol consumption elevates serum high-density lipoprotein cholesterol (HDL-C) concentration, one of the sources of the cardioprotective effect associated with alcohol consumption [9]. However, it is controversial why the increasing effect of alcohol consumption on HDL-C provides the cardioprotective effect associated with alcohol consumption. Japanese cohort study showed that extremely high HDL was associated with mortality of CVD [10].

Most short-term intervention studies (up to three weeks), including those in Japan, have suggested that moderate alcohol consumption (up to 60 g/day of alcohol) increases serum HDL-C; however, these studies have focused on men only or on both sexes [11]. The increasing effect of alcohol on HDL-C can differ according to gender, age, and alcohol consumption period because lipid and lipoprotein metabolisms and alcohol degradation capacities can differ depending on sex and age [12,13]. Serum HDL-C levels in women can be increased by regular exercise but decreased by habitual smoking and after menopause [14,15]. Interestingly, one longitudinal study in Korean women suggested that habitual moderate alcohol intake (about 60g of alcohol/week) was associated with a decrease in HDL-C, nevertheless, the sample size was small (n = approximately 20) [16]. Uncertainty remains about the association between long-term habitual alcohol consumption and serum HDL-C in women.

We sought to investigate the association between habitual alcohol consumption and changes in serum HDL-C in middle-aged Japanese women in a community-based 10-year cohort study using conventional statistical analyses. Furthermore, to investigate potential factors contributing to changes in HDL-C besides alcohol consumption (age, body weight, smoking, and other blood parameters), we also applied an explainable artificial intelligence (AI) analysis.

## Materials and methods

Study design

The National Database Study in the Kanto 7 Prefectures (NDB-K7Ps Study, conducted in Tokyo, Kanagawa, Saitama, Chiba, Ibaraki, Gunma, and Tochigi, Japan) was a composite multidisciplinary study to investigate the clinical factors primarily associated with cardiometabolic disease and metabolic syndrome, involving secondary use of annual health checkup data. Details of the study concept and design have been described elsewhere [17]. Since 2008, all Japanese people aged 40-74 years have been recommended to undergo a yearly itemized health checkup managed by Japan’s Ministry of Health, Labour, and Welfare (MHLW) [18]. After a rigorous review of our research project by the MHLW, our protocol was accepted in December 2020. We received digitally recorded anonymous data from the MHLW in July 2022. This study was conducted according to the guidelines of the Declaration of Helsinki and approved by the Institutional Review Board of the Ethics Committee of Japan Women's University (No. 513) and the MHLW (No. 1320).

Measurements and clinical parameters

The amount of alcohol consumption was evaluated with the following survey questions created by the MHLW [18]: “How much do you drink a day, in terms of glasses of refined sake? (A glass (180 mL) of refined sake is equivalent to a medium bottle (500 mL) of beer, 80 mL of shochu (alcohol content 35%), a glass (double, 60 mL) of whiskey, and two glasses (240 mL) of wine).” According to the response, we classified subjects into three groups: (1) Light drinkers, <23g (<180 mL) ethanol/day; (2) Moderate drinkers, 23-45g (180-360 mL) ethanol/day; or (3) Heavy drinker, ≥46 g (≥360 mL) ethanol/day. Drinking frequency was also evaluated with the survey question: “How often do you drink alcohol (e.g., sake, distilled spirits, beer, liquor)?”. According to the response, we classified subjects into three groups: (1) Everyday, (2) Occasional, or (3) Rarely or not at all (cannot drink). Body mass index (BMI) was calculated as body weight (kg) divided by the square of height (m). Serum HDL-C, low-density lipoprotein cholesterol (LDL-C), and triglyceride (TG) concentrations were measured automatically, mainly spectrophotometrically (using a direct, non-precipitation method) [18]. Low levels of serum HDL-C (<50 mg/dL) in females were one of the criteria for clinical diagnosis of the metabolic syndrome [19], whereas extremely high levels of serum HDL-C (≥90 mg/dL) had adverse effects on CVD mortality [10]. Therefore, low and high HDL-C were defined as serum HDL-C of <50 and ≥90 mg/dL, respectively.

Subjects

**Figure 1:**
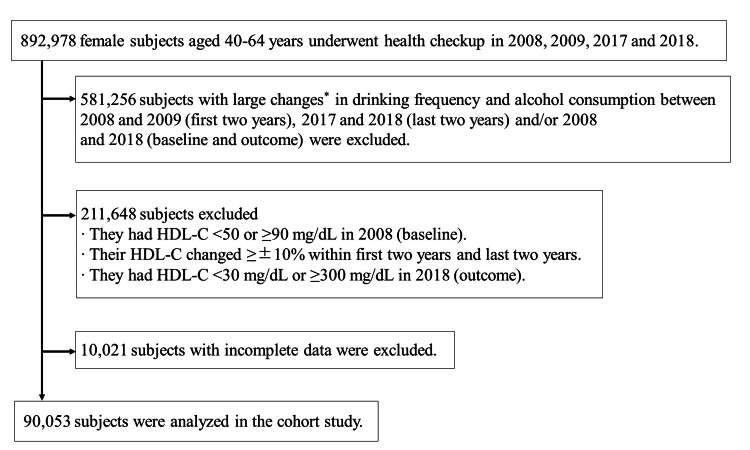
Exclusion criteria and subject disposition in the study ^*^Subjects whose drinking frequency or alcohol consumption changed ≥2 categories, for instance, whose alcohol consumption changed from <23 g to ≥46 g, or whose drinking frequency changed from “rarely or not at all” to “everyday”.

The exclusion criteria are shown in Figure 1. We initially reviewed data collected from 892,978 non-hospitalized women aged 40-64 years who underwent health check-ups in April 2008-March 2009 (2008), April 2009-March 2010 (2009), April 2017-March 2018 (2017) and April 2018-March 2019 (2018). To exclude subjects with large changes in alcohol consumption or drinking frequency during the period of the survey, subjects whose alcohol consumption and drinking frequency changed by ≥2 categories (for instance, alcohol consumption changed from <23 g to ≥46 g, or drinking frequency changed from “rarely or not at all” to “everyday”) between 2008 and 2009 (first two years), 2017 and 2018 (last two years) and/or 2008 and 2018 (baseline and outcome) were excluded. Additionally, subjects outside of the normal HDL-C range (<50 or ≥90 mg/dL) [10,19] at baseline and subjects with extremely low or high HDL-C (<30 mg/dL or ≥300 mg/dL) in 2018 were excluded. Moreover, to ensure the reproducibility of HDL-C levels, subjects with large changes (≥±10%) in HDL-C within the first two years, last two years, and/or baseline and outcome were excluded. Finally, subjects with incomplete data were excluded. A total of 90,053 subjects satisfied these criteria and were analyzed in the cohort study.

Statistical analysis

Data were expressed as means ± standard deviation or medians (interquartile range). We transformed the age groups (40-44, 45-49, 50-54, 55-59, 60-64, and 65-69 years) into substituted ages (S-age) corresponding to the median for each age group (42, 47, 52, 57, 62, and 67 years, respectively). Continuous and categorical variables were analyzed using analysis of co-variance adjusting for S-age. One-way analysis of variance with Dunnett’s multiple comparison test was used to examine the associations among the alcohol consumption categories. Logistic regression models were conducted to examine the associations between alcohol consumption and decrease/increase of ≥10 mg/dL or 10% in serum HDL-C compared with baseline HDL-C and outcome low/high HDL-C; these results are presented as odds ratios (ORs) and 95% confidence intervals (CIs). We adjusted for potential confounding factors (age, BMI, pharmacotherapy for dyslipidemia, smoking status, habitual exercise (≥30 min exercise per session, >2 times/week vs. less frequent exercise), baseline TG, LDL-C, ALT, γ-GTP and HbA1c; model 2), and baseline serum HDL-C which may most influence outcome HDL-C (model 3). These conventional statistical analyses were performed using the SAS-Enterprise Guide (SAS-EG 8.3) in SAS version 9.4 (SAS Institute, Cary, NC, USA). A two-tailed p<0.05 was considered significant.

AI analyses were performed using Prediction One (Sony Network Communications Inc., Tokyo, Japan), an explanatory AI analysis system with machine learning algorithms of extreme gradient boosting (XGBoost) and neural network algorithms [20]. The contributors to the prediction of a decrease of ≤10 mg/dL or 10% in serum HDL-C after 10 years were listed in order according to each feature’s importance, reflecting the relative contribution degree as a continuous value. To improve the generalization ability and prevent overfitting, cross-validation was automatically performed after dividing the total data into five divisions [21]. Previous studies showed that over threefold cross-validation improved the generalization ability and prevented overfitting [21]. In Prediction One, fivefold cross-validation was automatically performed after dividing the total data into test data sets and validation data sets [20], whose details are trade secrets and could not be provided. Total classification accuracy in the prediction model was assessed by the area under the receiver operating characteristic curve (AUC), with >74% and 63%-73% being considered good and standard predictive models, respectively [20].

## Results

Table 1 shows the baseline clinical characteristics of participants. The proportion of heavy drinkers was lowest among the three groups (4.3% of all subjects). Heavy drinkers showed the highest serum HDL-C in the three groups (*p *< 0.0001). Additionally, heavy drinkers were youngest and showed the highest BMI, diastolic blood pressure (DBP), TG, aspartate aminotransferase (AST), alanine aminotransferase (ALT), γ-glutamyl transferase (γ-GTP) and TG/HDL-C, as well as the lowest systolic blood pressure (SBP), LDL-C, LDL-C/HDL-C and glycated hemoglobin (HbA_1c_) (all* p* < 0.0001). Similarly, heavy drinkers showed the highest proportion of current smokers; occasional and everyday drinkers, however, showed the lowest proportions of the other categorical variables (all p < 0.0001).

**Table 1:** Baseline clinical characteristics of participants Data are presented as mean ± standard deviation (SD), median (interquartile range; IQ) for TG, or n (%). Statistical significance of difference between alcohol consumption categories was compared using analysis of co-variance adjusting for S-age. Analysis of co-variance for categorical variables was conducted after conversion to continuous variables. Significant differences were observed in all continuous and categorical variables between alcohol consumption categories (p* *< 0.0001). ^*^Regular exercise defined as ≥ 30 minutes at least twice a week. ALT: alanine aminotransferase; AST: aspartate aminotransferase; BMI: body mass index; DBP: diastolic blood pressure; GTP: γ-glutamyl transferase; HbA_1c_: glycated hemoglobin HDL-C: high-density lipoprotein cholesterol; LDL-C: low-density lipoprotein cholesterol; S-age: substituted age; SBP: systolic blood pressure; TG: triglycerides.

Alcohol consumption categories	All	Light (<23g/day)	Moderate (23-45g/day)	Heavy (≥46g/day)
N (%)	90,053	66,859 (74.2)	19,339 (21.5)	3,855 (4.3)
S-age (years old)	53.0 ± 7.4	53.6 ± 7.4	51.6 ± 7.3	49.1 ± 6.6
BMI (kg/m^2^)	21.9 ± 3.1	21.9 ± 3.1	22.0 ± 3.2	22.3 ± 3.3
SBP (mmHg)	120.3 ± 17.2	120.5 ± 17.2	120.0 ± 17.2	119.7 ± 17.2
DBP (mmHg)	73.4 ± 11.0	73.3 ± 11.0	73.6 ± 11.1	74.1 ± 11.6
TG (IQ)(mg/dL)	77 (57-105)	77 (57-105)	75 (56-104)	78 (57-110)
HDL-C (mg/dL)	69.8 ± 10.3	69.3 ± 10.3	70.9 ± 10.1	71.8 ± 10.2
LDL-C (mg/dL)	125.6 ± 30.5	127.3 ± 30.1	121.9 ± 30.8	115.2 ± 31.3
LDL-C/ HDL-C (ratio)	1.9 ± 0.6	1.9 ± 0.6	1.8 ± 0.6	1.7 ± 0.6
TG/ HDL-C (ratio)	1.3 ± 0.8	1.3 ± 0.8	1.3 ± 0.8	1.4 ± 1.0
AST (U/L)	21.3 ± 6.8	21.3 ± 6.6	21.1 ± 7.1	21.4 ± 8.1
ALT (U/L)	18.4 ± 9.9	18.5 ± 9.8	18.2 ± 10.1	18.4 ± 10.4
γ-GTP (U/L)	24.8 ± 21.9	23.6 ± 19.6	27.3 ± 25.7	33.3 ± 33.3
HbA_1c _(%)	5.5 ± 0.5	5.5 ± 0.5	5.4 ± 0.5	5.3 ± 0.5
Pharmacotherapy				
Hypertension, n (%)	5,942 (9.9)	4,325 (10.0)	1,356 (9.9)	261 (9.2)
Diabetes, n (%)	552 (0.9)	441 (1.0)	94 (0.7)	17 (0.6)
Dyslipidemia, n (%)	632 (1.1)	3,572 (8.2)	791 (5.8)	84 (3.0)
Cardiovascular disease, n (%)	1,097 (1.9)	813 (1.9)	245 (1.8)	39 (1.4)
Current smokers, n (%)	5,777 (9.6)	2,825 (6.5)	2,119 (15.5)	833 (29.4)
Regular exercise, n (%) ^*^	16,018 (27.3)	11,869 (27.8)	3,496 (26.2)	653 (23.8)
Drinking frequency				
Rarely or not at all , n (%)	18,939 (31.6)	18,403 (42.3)	478 (3.5)	58 (2.0)
Occasionally, n (%)	27,329 (45.5)	18,499 (42.5)	7,512 (54.8)	1,318 (46.4)
Every day, n (%)	13,759 (22.9)	6,578 (15.1)	5,717 (41.7)	1,464 (51.5)

Table 2 shows BMI and HDL-C levels at baseline (2008) and outcome (2018) according to ΔHDL-C categories and alcohol consumption categories in unadjusted data. According to ΔHDL-C, calculated by subtracting HDL-C concentration in 2008 from that in 2018, we categorized subjects into three groups: ΔHDL-C ≤10%, -10% to 10% and ≥10%. Baseline age and BMI (both in 2008 and 2018) were higher in the ΔHDL-C ≤10% group than the other groups. Outcome BMI was higher than baseline BMI in ΔHDL-C ≤10% and -10% to 10%, except for ΔHDL-C ≥10% group. The ΔHDL-C ≤10% group showed the highest baseline serum HDL-C concentrations in the three ΔHDL groups. In ΔHDL-C ≤10% and ≥10% groups, heavy drinkers showed the highest baseline HDL-C concentration of the three drinker groups, except for ΔHDL-C -10~10% group.

**Table 2:** BMI and HDL-C levels at baseline (2008) and outcome (2018) according to ΔHDL-C categories and alcohol consumption categories. Data are presented as mean ± SD.

ΔHDL-C	ΔHDL-C ≤10% (n = 16,124; 17.9% of all)						ΔHDL-C -10~10% (n = 50,902; 56.5% of all)						ΔHDL-C ≥10% (n = 23,027; 25.6% of all)					
Alcohol consumption	Light		Moderate		Heavy		Light		Moderate		Heavy		Light		Moderate		Heavy	
Year	2008	2018	2008	2018	2008	2018	2008	2018	2008	2018	2008	2018	2008	2018	2008	2018	2008	2018
N	11,728		3,615		781		38,190		10,696		2,016		16,941		5,028		1,058	
Baseline age	54.4 ± 7.0		52.6 ±7.0		49.7 ± 6.6		53.9 ± 7.3		51.7 ± 7.3		49.3 ± 6.7		52.5 ± 7.7		50.4 ± 7.3		48.2 ± 6.4	
BMI	22.2 ± 3.0	23.3 ± 3.4	22.4 ± 3.0	23.6 ± 3.6	22.5 ± 3.1	23.9 ± 3.7	21.9 ± 3.1	22.3 ± 3.4	22.0 ± 3.1	22.5 ± 3.5	22.4 ± 3.3	22.8 ± 3.7	21.6 ± 3.1	21.3 ± 3.3	21.8 ± 3.3	21.6 ± 3.4	22.0 ± 3.6	21.7 ± 3.6
HDL-C	71.3 ± 10.1	59.7 ± 9.1	72.7 ± 9.9	60.7 ± 9.1	73.4 ± 9.8	60.8 ± 9.3	69.6 ± 10.3	69.6 ± 10.8	71.2 ± 10.1	71.2 ± 10.7	71.7 ± 10.3	71.7 ± 10.9	67.4 ± 10.2	80.3 ± 12.8	69.1 ± 10.2	82.4 ± 13.1	70.7 ± 10.2	84.9 ± 13.3

**Figure 2:**
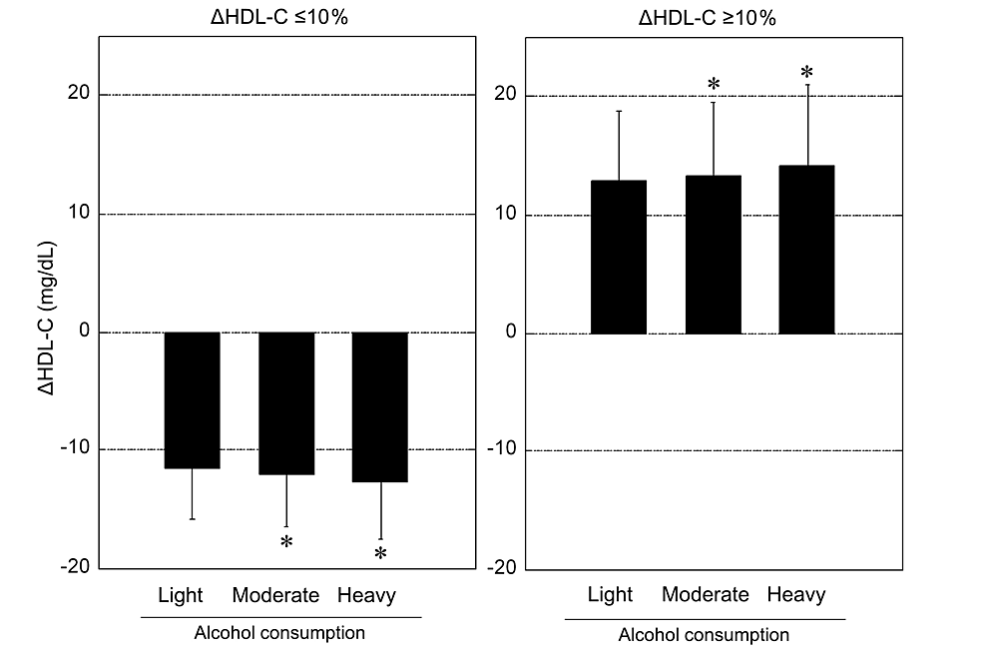
ΔHDL-C levels according to ΔHDL-C categories (ΔHDL-C ≤10% and ΔHDL-C ≥10%) and alcohol consumption categories. Data are presented as mean ± SD. Asterisks indicate the significant difference versus light drinker group (*p* < 0.0001, determined by one-way analysis of variance with Dunnett’s multiple comparisons test).

Figure 2 shows ΔHDL-C levels according to ΔHDL-C categories (ΔHDL-C ≤10% and ΔHDL-C ≥10%) and alcohol consumption categories. In the ΔHDL-C ≤10% group, moderate and heavy drinkers showed a larger ΔHDL-C toward decreasing serum HDL-C than light drinkers (*p* < 0.0001). Similarly, in ΔHDL-C ≥10% group, moderate and heavy drinker groups showed a larger ΔHDL-C toward increasing serum HDL-C than light drinkers (*p* < 0.0001). 

Table 3 shows the ORs and 95% CIs of alcohol consumption for decrease/increase in serum HDL and low/high HDL. After 10 years, 11.3% and 17.9% of subjects had decreases in serum HDL-C of ≥10 mg/dL and ≥10%, respectively; 17.3% and 25.6% of subjects showed increases in serum HDL-C of ≥10 mg/dL and ≥10%, respectively. After 10 years, 3.5% of subjects had low HDL-C and 8.7% of subjects had high HDL-C. After adjustment for potential confounding factors and baseline serum HDL-C, ORs for a decrease in serum HDL-C were significantly higher in moderate and heavy drinkers than light drinkers (reference level of 1.00): 1.15 and 1.23 (95% CIs 1.08-1.22 and 1.10-1.39), respectively, for ≥ 10 mg/dL, and 1.09 and 1.23 (1.04-1.15 and 1.11-1.36) for ≥ 10%. Occasional and everyday drinking were not associated with decreases in HDL-C compared with subjects in the rarely or not at all group (reference level of 1.00), with ORs of 0.98 (95% CI 0.93-1.04) and 1.04 (95% CI 0.97-1.12) for ≥10 mg/dL, and 1.01 (95% CI 0.97-1.06) and 1.03 (95% CI 0.97-1.09) for ≥10% (data not shown). ORs for an increase in serum HDL-C after 10 years were significantly higher in heavy drinkers than in light drinkers in unadjusted model 1 (1.19 (95% CI 1.10-1.30) for ≥10 mg/dL, 1.12 (1.04-1.20) for ≥10%), but were not significantly different from those in light drinkers in model 3 after adjustment for potential confounding factors and baseline serum HDL-C (0.99 (95% CI 0.90-1.10) for ≥10 mg/dL, 1.02 (0.93-1.11) for ≥10%). The OR for low HDL-C was significantly higher in heavy drinkers than light drinkers only in model 3 (1.43 (95% CI 1.13-1.81)), whereas ORs for high HDL-C were significantly higher in heavy drinkers than light drinkers in all three models at 1.71 (95% CI 1.55-1.88), 1.50 (95% CI 1.33-1.70) and 1.16 (95% CI 1.01-1.33).

**Table 3:** ORs (95% CIs) of alcohol consumption for decrease/increase in serum HDL-C and low/high HDL-C ^*p < 0.05; **p < 0.01; ***p < 0.0001^ Model 1: Unadjusted. Model 2: Adjusted for age, BMI, pharmacotherapy for dyslipidemia, smoking status, habitual exercise (≥30 min exercise per session, >2 times/week vs. less frequent exercise), baseline TG, LDL-C, ALT, γ-GTP and HbA_1c_. Model 3: Model 2 plus adjustment for baseline HDL-C.

Alcohol consumption	Light (n = 66,859)	Moderate (n = 19,339)	Heavy (n = 3,855)
Decrease of ≥10 mg/dL (n = 10,189 / 11.3% of all)			
n (% in each group)	7,230 (10.8)	2,433 (12.6)	526 (13.6)
Model 1	1 (ref)	1.19 (1.13-1.25) ^***^	1.30 (1.19-1.43) ^***^
Model 2		1.18 (1.11-1.26) ^***^	1.36 (1.21-1.53) ^***^
Model 3		1.15 (1.08-1.22) ^***^	1.23 (1.10-1.39) ^**^
Decrease of ≥10% (n = 16,124 / 17.9% of all)			
n (% in each group)	11,728 (17.5)	3,615 (18.7)	781 (20.3)
Model 1	1 (ref)	1.08 (1.04-1.13) ^**^	1.19 (1.10-1.30) ^***^
Model 2		1.11 (1.05-1.17) ^***^	1.29 (1.17-1.43) ^***^
Model 3		1.09 (1.04-1.15) ^**^	1.23 (1.11-1.36) ^***^
Increase of ≥10 mg/dL (n = 15,611 / 17.3% of all)			
n (% in each group)	11,420 (17.1)	3,430 (17.7)	761 (19.7)
Model 1	1 (ref)	1.05 (1.00-1.09) ^*^	1.19 (1.10-1.30) ^***^
Model 2		0.96 (0.91-1.01)	0.98 (0.89-1.08)
Model 3		0.96 (0.92-1.02)	0.99 (0.90-1.10)
Increase of ≥10% (n = 23,027 / 25.6% of all)			
n (% in each group)	16,941 (25.3)	5,028 (26.0)	1,058 (27.4)
Model 1	1 (ref)	1.04 (1.00-1.07)	1.12 (1.04-1.20) ^**^
Model 2		0.97 (0.93-1.01)	0.96 (0.88-1.05)
Model 3		0.99 (0.94-1.03)	1.02 (0.93-1.11)
Low HDL-C (<50 mg/dL) (n = 3,121 / 3.5% of all)			
n (% in each group)	2,406 (3.6)	584 (3.0)	131 (3.4)
Model 1	1 (ref)	0.83 (0.76-0.91) ^**^	0.94 (0.79-1.13)
Model 2		0.97 (0.86-1.09)	0.98 (0.78-1.23)
Model 3		1.11 (0.98-1.25)	1.43 (1.13-1.81) ^**^
High HDL-C (≥90 mg/dL) (n = 7,823 / 8.7% of all)			
n (% in each group)	5,374 (8.0)	1,949 (10.1)	500 (13.0)
Model 1	1 (ref)	1.28 (1.21-1.35) ^***^	1.71 (1.55-1.88)^ ***^
Model 2		1.13 (1.06-1.21) ^**^	1.50 (1.33-1.70)^ ***^
Model 3		1.02 (0.95-1.10)	1.16 (1.01-1.33) ^*^

Table 4 shows the contributors to the prediction of decrease in serum HDL-C in order of importance obtained by AI analysis. The AUC was 68.4%. In the prediction model, most positive contributor of decrease of ≤10 mg/dL or 10% in serum HDL-C after 10 years was baseline high serum HDL-C (feature importance: 0.1461). Alcohol consumption was the eighth contributor to a decrease of ≥10 mg/dL in serum HDL-C, following baseline high HDL-C (≥77 mg/dL), high LDL-C (≥133 mg/dL), high BMI (≥23.1 kg/m^2^), pharmacotherapy for dyslipidemia, high TG (≥70 mg/dL), subject age 44-64 years, and current smoking. Among the alcohol consumption groups, heavy alcohol consumption was the most positive contributor to a decrease of ≥10 mg/dL in serum HDL-C. The order of contributors to a decrease of ≥10% in serum HDL-C was essentially the same.

**Table 4:** Parameters contributing to the prediction of decrease in serum HDL-C Contribution direction/order and range of variables were automatically generated by machine learning. The feature importance reflects the contribution degree as a continuous value. ^*^Feature importance of each variable itself. ^**^Feature importance in the detail range of each variable. Cross-validation was automatically performed after the total data were divided into five divisions.

Decrease of ≥10 mg/dL										
Contribution order	Variables	Feature importance^*^	Contribution direction/order				Range			Feature importance for each range^**^
1	HDL-C (mg/dL)	0.1461	Positive	1	86-89			0.0828		
				2	81-86			0.0726		
				3	77-81			0.0467		
			Negative	1	50-56			0.0944		
				2	56-61			0.0630		
				3	61-65			0.0450		
2	LDL-C (mg/dL)	0.0394	Positive	1	173-298			0.0193		
				2	154-173			0.0173		
				3	133-142			0.0110		
			Negative	1	30-88			0.0277		
				2	88-100			0.0192		
				3	100-109			0.0086		
3	BMI (kg/m^2^)	0.0344	Positive	1	24.2-26.0			0.0149		
				2	23.1-24.2			0.0128		
				3	26.0-52.1			0.0103		
			Negative	1	11.9-18.5			0.0276		
				2	18.5-19.4			0.0198		
				3	20.1-20.8			0.0085		
4	Pharmacotherapy for Dyslipidemia	0.0311	Positive	1	yes			0.0219		
			Negative	1	no			0.0056		
5	TG (mg/dL)	0.0254	Positive	1	102-121			0.0134		
				2	121-160			0.0086		
				3	70-79			0.0081		
			Negative	1	30-45			0.0188		
				2	45-54			0.0106		
				3	54-62			0.0079		
6	Age	0.0210	Positive	1	44-54			0.0152		
				2	50-59			0.0132		
				3	60-64			0.0049		
			Negative	1	40-44			0.0072		
				2	40-49			0.0063		
7	Current smoking	0.0175	Positive	1		yes			0.0198	
			Negative	1		no			0.0060	
8	Alcohol consumption (g ethanol/session)	0.0158	Positive	1		≥46g			0.0139	
				2		<23g, 23-45g			0.0076	
			Negative	1		<23g			0.0052	
9	γ-GTP	0.0127								
10	Drinking frequency	0.0121								
Total classification accuracy (AUC)		68.4%								

Decrease of ≥10%						
Contribution order	Variables	Feature importance^*^	Contribution direction/order		Range	Feature importance for each range^**^
1	HDL-C (mg/dL)	0.1064	Positive	1	86-89	0.0608
				2	81-86	0.0501
				3	77-81	0.0372
			Negative	1	50-56	0.0751
				2	56-61	0.0391
				3	61-65	0.0305
2	LDL-C (mg/dL)	0.0558	Positive	1	173-298	0.0297
				2	154-173	0.0283
				3	142-154	0.0161
			Negative	1	30-88	0.0363
				2	88-100	0.0252
				3	100-109	0.0096
3	BMI (kg/m^2^)	0.0510	Positive	1	24.2-26.0	0.0231
				2	23.1-24.2	0.0214
				3	22.2-23.1	0.0170
			Negative	1	11.9-18.5	0.0423
				2	18.5-19.4	0.0253
				3	19.4-20.1	0.0112
4	TG (mg/dL)	0.0377	Positive	1	102-121	0.0201
				2	121-160	0.0187
				3	70-79	0.0121
			Negative	1	30-45	0.0243
				2	45-54	0.0138
				3	54-62	0.0106
5	Dyslipidemia	0.0369	Positive	1	yes	0.0301
			Negative	1	no	0.0060
6	Age	0.0311	Positive	1	45-54	0.0244
				2	50-59	0.0175
				3	60-64	0.0059
			Negative	1	40-44	0.0121
				2	40-49	0.0084
7	Current smoking	0.0195	Positive	1	yes	0.0198
			Negative	1	no	0.0063
8	Alcohol consumption (g ethanol/session)	0.0185	Positive	1	≥46g	0.0204
				2	<23g, 23-45g	0.0076
			Negative	1	<23g	0.0048
9	Drinking frequency	0.0171				
10	AST	0.0168				
Total classification accuracy (AUC)		61.9%				

Furthermore, we also investigated the association between habitual alcohol consumption and changes in serum HDL-C in Japanese middle-aged men. A total of 186,624 subjects satisfied the criteria (Figure 1) and were analyzed in the cohort study. Average age of male subject was 50.1 ± 6.8; average BMI was 23.7 ± 3.0 kg/m2; 18,122 (31.4%) participants were current smokers at baseline (data not shown). Among middle-aged men, moderate-to-heavy alcohol consumption was significantly associated with a decrease of ≤10 mg/dL in HDL-C; however, it was also associated with increases of ≥10 mg/dL and 10% in HDL-C and high HDL-C (Table 5).

**Table 5:** ORs (95% CIs) of alcohol consumption for decrease/increase in HDL-C and low/high HDL-C in men ^*p < 0.05; **p < 0.01; ***p < 0.0001^

Alcohol consumption	Light (n = 65,860)	Moderate (n = 74,027)	Heavy (n = 46,737)	
Decrease of HDL-C ≥10 mg/dL (n = 18,208 / 9.8% of all)				
n (% in each group)	5,344 (8.1)	7,526 (10.2)	5,338 (11.4)	
Model 1	1 (ref)	1.28 (1.24-1.33)^ ***^	1.46 (1.40-1.52)^ ***^	
Model 2		1.20 (1.14-1.26)^ ***^	1.45 (1.38-1.52) ^***^	
Model 3		1.07 (1.02-1.13)^ **^	1.10 (1.04-1.16) ^**^	
Decrease of HDL-C ≥10% (n = 36,595 / 19.6% of all)				
n (% in each group)	12,172 (18.5)	14,741 (19.9)	9,682 (20.7)	
Model 1	1 (ref)	1.10 (1.07-1.13)^ ***^	1.15 (1.12-1.19)^ ***^	
Model 2		1.08 (1.04-1.11)^ ***^	1.17 (1.12-1.21)^ ***^	
Model 3		1.02 (0.99-1.06)	1.02 (0.98-1.06)	
Increase of HDL-C ≥10 mg/dL (n = 27,892 / 14.9% of all)				
n (% in each group)	8,865 (13.5)	11,319 (15.3)	7,708 (16.5)	
Model 3	1 (ref)	1.09 (1.05-1.13)^ ***^	1.17 (1.12-1.22)^ ***^	
Increase of HDL-C ≥10% (n = 50,487 / 27.0% of all)				
n (% in each group)	17,288 (26.2)	20,100 (27.2)	13,090 (28.0)	
Model 3	1 (ref)	1.06 (1.02-1.09)^ **^	1.14 (1.10-1.18)^ ***^	
Low HDL-C (<40 mg/dL) (n = 4,707 / 2.5% of all)				
n (% in each group)	2,023 (3.1)	1,692 (2.3)		992 (2.1)
Model 3	1 (ref)	0.99 (0.90-1.08)		1.01 (0.91-1.12)
High HDL-C (≥90 mg/dL) (n = 5,112 / 2.7% of all)				
n (% in each group)	1,260 (1.9)	2,132 (2.9)		1,720 (3.7)
Model 3	1 (ref)	1.05 (0.96-1.15)		1.14 (1.03-1.26) ^*^

## Discussion

This cohort study suggested that habitual moderate-to-heavy alcohol consumption decreased serum HDL-C among over 90,000 middle-aged Japanese women whose alcohol consumption and drinking frequency were generally consistent over 10 years. While moderate-to-heavy alcohol consumption did not increase serum HDL-C in women in this study, heavy alcohol consumption was significantly associated with both low HDL-C and high HDL-C in women (Table 3). Although heavy alcohol consumption was associated with decreased serum HDL-C compared with baseline HDL-C in women, several subjects may have been categorized as having high HDL-C at outcome because these women had a high HDL-C level on average. In middle-aged men, habitual alcohol consumption may decrease serum HDL-C in part; however, there may be an overall tendency for serum HDL-C to increase with habitual alcohol consumption (Table 5).

Most short-term (up to three weeks) intervention studies for middle-aged subjects that included both sexes or men only, including in Japan, showed that light-to-moderate alcohol intake (up to 60 g of alcohol/day, similar to our study) increased serum HDL-C [11]. However, a previous Korean study showed that long-term habitual alcohol consumption (approximately 60 g of alcohol/week and ≥10 years) caused a significant decrease in serum HDL-C in middle-aged women (35-62 years old) [16]. Because we investigated subjects who maintained their drinking habits over 10 years, our results reflect the effect of long-term habitual alcohol consumption on serum HDL-C, as in the Korean study. Although the Korean study was based on a small sample (approximately 20 subjects), it may nonetheless support our results. The effect of alcohol consumption on serum HDL-C may differ between men and women, as well as between short-term and long-term consumption.

While a large American cohort study suggested that serum HDL-C was increased as drinking frequency was increased in both men and women [22], drinking frequency was not significantly associated with changes in serum HDL-C in this study. However, the American study more finely categorized subjects in terms of drinking frequency than did our study, with categories of <1, 1-2, 3-4, and 5-7 days/week. Further investigations are needed to confirm these findings in greater detail regarding drinking frequency, and additionally to categorize subjects based on both drinking frequency and alcohol consumption.

The mechanisms underlying the decreasing effect of habitual moderate-to-heavy alcohol consumption on serum HDL-C observed in this study remain unclear. Most interventional and observational studies have suggested that the underlying mechanism may involve primary proteins and enzymes such as cholesterol ester transfer protein (CETP), lipoprotein lipase (LPL), paraoxonase (PON), and cholesterol efflux capacity (CEC). Concretely, alcohol intake may elevate serum HDL-C via inhibition of CETP activity and enhancement of LPL and PON activity and CEC [11]. The aforementioned Korean study suggests that alcohol consumption decreases HDL-C quality and functionality; specifically, habitual drinkers had lower serum apolipoprotein A-I and PON activity and smaller particle size of HDL subfraction HDL3 compared with the non-drinker group [16]. Originally, women had greater HDL-C concentration and lower LDL-C, very low-density lipoprotein cholesterol, and TG than age-matched men. Additionally, women have a greater HDL apolipoprotein A-I and A-II synthesis rate and larger HDL particles than men [23]. These sex differences in lipid and lipoprotein metabolisms and particle sizes are likely to account for at least part of the cardioprotective effect of women. However, it is unclear why habitual alcohol consumption affected apolipoprotein A-I and particle size of HDL in only women such as above Korean study [16]. Unfortunately, in our study, the mechanisms leading to decreased HDL-C in middle-aged women remain unclear because these proteins and enzyme activities are not regularly measured in Japanese health check-ups. Further studies are needed to elucidate the biological mechanisms in the sex differences of the effect of alcohol consumption.

The results shown in Table 2 and Figure 2 suggest that heavy drinkers with decreased HDL-C may have the highest BMI and baseline HDL-C and greatest decreases in HDL-C at outcome. Furthermore, AI analysis showed that alcohol-related decreases in HDL-C may be modified by concomitant factors, such as baseline high HDL-C, high LDL-C, high BMI, pharmacotherapy for dyslipidemia, high TG, subject age, and smoking status (Table 4). Alcohol intake is known to be associated with an elevated risk of metabolic syndrome [24], a cluster of metabolic factors including abdominal obesity, high blood pressure, impaired fasting glucose, and high TG and low HDL-C levels. Additionally, smoking is a crucial risk factor for metabolic syndrome and CVD [25,26]. As noted above, habitual drinking in women may be linked to unhealthy lifestyle factors inducing obesity and dyslipidemia. Our study and the Korean study [16] showed the decreasing effect of alcohol intake on HDL-C in long-term cohort studies in women, unlike the findings of short-term intervention studies, which may be related to several alcohol-involved concomitant factors inducing obesity and dyslipidemia, rather than physiological differences between the sexes. Although the differences in biological reaction between short-term and long-term alcohol consumption such as serum apolipoprotein A-I, PON activity, and particle size of HDL also remain unclear, these differences may occur due to several biological changes related to obesity and dyslipidemia caused by long-term alcohol consumption.

In our AI analysis, the reasons for pharmacotherapy for dyslipidemia being a positive contributor to a decrease in HDL-C were unclear. This may have been a result of reverse causality because patients with dyslipidemia had lower baseline HDL-C and greater decreases in HDL-C than those without dyslipidemia (data not shown). Compared with patients without dyslipidemia, patients with dyslipidemia may have poor lipid control even with pharmacotherapy.

A previous study has suggested that postmenopausal women have higher serum LDL-C and TG and lower serum HDL-C than premenopausal women. Concretely, in postmenopausal women, HDL2 levels decrease whereas HDL3 levels increase (HDL2 is more antiatherogenic than HDL3). Additionally, LPL activity slightly increases through menopause, whereas CETP activity does not change [15]. These changes in lipid and lipoprotein metabolism through menopause may be concomitant changes in total body fat, body fat distribution, and insulin sensitivity that accompany menopause [23]. Whereas, in a short-term clinical trial, moderate alcohol consumption increased serum HDL-C levels and stimulated cellular CEC in postmenopausal women [27]. Our dataset included no information regarding pre- or postmenopausal status. However, decreasing effects of alcohol intake on serum HDL-C were apparent when the subject age was limited to those over 50 years old (data not shown). Additionally, in this study, moderate-to-heavy alcohol consumption was positively associated with high TG in both sexes and high LDL-C in women only (data not shown). Although the finding of high TG via alcohol intake agrees with most previous reports in both sexes and men alone [9,22], a previous meta-analysis of intervention studies in both sexes, including pre- and postmenopausal women, suggested that alcohol intake decreased or had no effect on serum LDL-C [11], in contrast with our results. Further investigations about the effects of alcohol intake on serum lipids are needed to target pre- and postmenopausal women.

The strength of our study is its focus on over 90,000 middle-aged Japanese women who had unchanged alcohol habits for 10 years and baseline normal HDL-C. However, several limitations should be mentioned in our study. First, we used self-report questionnaires which may introduce potential self-report bias in alcohol consumption data. However, we reduced self-report bias by excluding subjects with large changes in alcohol consumption or drinking frequency between 2008 and 2009 (first two years), 2017 and 2018 (last two years), and/or 2008 and 2018 (baseline and outcome). Second, detailed information regarding menopausal status was unavailable; serum HDL-C and other lipid concentrations may be affected by menopausal status, the period after menopause, and whether to take hormone replacement therapy [23]. Third, we were unable to categorize subjects by drinking frequency because of the relatively small sample size in the heavy drinker group. Fourth, detailed information about beverage type was unavailable; men and women may have different preferences for alcoholic beverages [28], and there are reported differences in the changes in HDL-C and other lipids between beer, wine, and hard liquor consumption [29]. Fifth, the reference in our logistic regression models was light drinkers (<23 g of alcohol/day) because we had no available information about non-drinkers. Sixth, although heavy drinkers are more likely to develop severe liver disease, in which the synthesis of HDL in the liver decreases [30], patients with chronic liver failure and hepatic cirrhosis were not excluded from this study. However, adjustment for γ-GTP and ALT did not change the relationship between alcohol consumption and HDL-C. Finally, the mechanisms underlying the decrease in HDL-C via alcohol consumption were unclear because detailed information about serum HDL subfraction, apolipoproteins, and enzyme activities was also unavailable, with measurement of these factors being unfeasible in a large population. Therefore, additional studies are needed to address these limitations, enhance the generalizability of our results, and elucidate the underlying mechanisms.

## Conclusions

We aimed to investigate the association between habitual alcohol consumption and changes in serum HDL-C in middle-aged Japanese women in a community-based 10-year cohort study. Although unadjusted data showed that moderate-to-heavy alcohol consumption may both increase and decrease serum HDL-C levels after 10 years, after adjustment for potential cofounding factors, habitual moderate-to-heavy alcohol consumption in middle-aged women may cause a significant decrease in serum HDL-C after 10 years, but not cause a significant increase in serum HDL-C. Further analysis after adjustment for baseline serum HDL-C level showed the same results. As AI analysis showed, the decreasing effect of alcohol intake on serum HDL-C in long-term cohort studies in women may be modified by several alcohol-involved concomitant factors inducing obesity and dyslipidemia, such as high BMI, high TG, high LDL-C and smoking status. Our results differ from the increasing effect on serum HDL-C of most short-term intervention studies for both sexes or men only, one of the sources of the cardioprotective effect associated with alcohol consumption. Further investigation of detailed information about beverage type, habitual food and nutrition intake, and menopausal status is needed to confirm our findings.

## Appendices

**Table 6:** STROBE Statement_Checklist of items that should be included in reports of cohort studies *Give information separately for exposed and unexposed groups. Note: An Explanation and Elaboration article discusses each checklist item and gives methodological background and published examples of transparent reporting. The STROBE checklist is best used in conjunction with this article (freely available on the Web sites of PLoS Medicine at http://www.plosmedicine.org/, Annals of Internal Medicine at http://www.annals.org/, and Epidemiology at http://www.epidem.com/). Information on the STROBE Initiative is available at http://www.strobe-statement.org.

	Item No	Recommendation	Relevant text from manuscript (PDF Page No.)
Title and abstract	1	(a) Indicate the study’s design with a commonly used term in the title or the abstract	Title (Page 1)
		(b) Provide in the abstract an informative and balanced summary of what was done and what was found	Abstract (Page 1)
Introduction			
Background/rationale	2	Explain the scientific background and rationale for the investigation being reported	Introduction (Page 1-2)
Objectives	3	State specific objectives, including any prespecified hypotheses	Introduction (Page 1-2)
Methods			
Study design	4	Present key elements of study design early in the paper	Material and Methods (Page 2-3)
Setting	5	Describe the setting, locations, and relevant dates, including periods of recruitment, exposure, follow-up, and data collection	Material and Methods (Page 2-3)
Participants	6	(a) Give the eligibility criteria, and the sources and methods of selection of participants. Describe methods of follow-up	Material and Methods (Page 2-3), Figure 1
		(b) For matched studies, give matching criteria and number of exposed and unexposed	—
Variables	7	Clearly define all outcomes, exposures, predictors, potential confounders, and effect modifiers. Give diagnostic criteria, if applicable	Material and Methods (Page 2-3)
Data sources/ measurement	8*	For each variable of interest, give sources of data and details of methods of assessment (measurement). Describe comparability of assessment methods if there is more than one group	Material and Methods (Page 2-3)
Bias	9	Describe any efforts to address potential sources of bias	Material and Methods (Page 2-3)
Study size	10	Explain how the study size was arrived at	Material and Methods (Page 2-3)
Quantitative variables	11	Explain how quantitative variables were handled in the analyses. If applicable, describe which groupings were chosen and why	Material and Methods (Page 2-3)
Statistical methods	12	(a) Describe all statistical methods, including those used to control for confounding	Material and Methods (Page 2-3)
		(b) Describe any methods used to examine subgroups and interactions	—
		(c) Explain how missing data were addressed	Material and Methods (Page 2-3)
		(d) If applicable, explain how loss to follow-up was addressed	Material and Methods Page (2-3), Figure 1
		(e) Describe any sensitivity analyses	—
Results			
Participants	13*	(a) Report numbers of individuals at each stage of study—eg numbers potentially eligible, examined for eligibility, confirmed eligible, included in the study, completing follow-up, and analysed	Material and Methods (Page 2-3), Figure 1
		(b) Give reasons for non-participation at each stage	Material and Methods (Page 2-3), Figure 1
		(c) Consider use of a flow diagram	Figure 1
Descriptive data	14*	(a) Give characteristics of study participants (eg demographic, clinical, social) and information on exposures and potential confounders	Results(Page 3-10), Table 1
		(b) Indicate number of participants with missing data for each variable of interest	—
		(c) Summarise follow-up time (eg, average and total amount)	Material and Methods (Page 2-3)
Outcome data	15*	Report numbers of outcome events or summary measures over time	Results (Page 3-10)
Main results	16	(a) Give unadjusted estimates and, if applicable, confounder-adjusted estimates and their precision (eg, 95% confidence interval). Make clear which confounders were adjusted for and why they were included	Results (Page 3-10), Table 3 and 5
		(b) Report category boundaries when continuous variables were categorized	Methods (Page 2-3), Table 3-5
		(c) If relevant, consider translating estimates of relative risk into absolute risk for a meaningful time period	—
Other analyses	17	Report other analyses done—eg analyses of subgroups and interactions, and sensitivity analyses	Results (Page 3-10)
Discussion			
Key results	18	Summarise key results with reference to study objectives	Discussion (page 11)
Limitations	19	Discuss limitations of the study, taking into account sources of potential bias or imprecision. Discuss both direction and magnitude of any potential bias	Discussion (page 11)
Interpretation	20	Give a cautious overall interpretation of results considering objectives, limitations, multiplicity of analyses, results from similar studies, and other relevant evidence	Discussion (page 11)
Generalisability	21	Discuss the generalisability (external validity) of the study results	Discussion (page 12)
Other information			
Funding	22	Give the source of funding and the role of the funders for the present study and, if applicable, for the original study on which the present article is based	Disclosures (page 12)
